# Incidence and Risk Factors of Bilateral Herpetic Keratitis: 2022 Update

**DOI:** 10.3390/tropicalmed7060092

**Published:** 2022-06-07

**Authors:** Stergios K Chaloulis, Georgios Mousteris, Konstantinos T Tsaousis

**Affiliations:** Ophthalmology Department, General Hospital of Volos, 134, Polymeri Str., 38221 Volos, Greece; stergioshaloulis@yahoo.gr (S.K.C.); trilosgate7@hotmail.com (G.M.)

**Keywords:** Herpes Simplex Virus, bilateral herpetic keratitis, disciform keratitis, polymerase chain reaction, acyclovir-resistant HSV

## Abstract

Simultaneously occurring bilateral herpetic keratitis is a rare clinical manifestation of ocular herpes. Immunocompromised patients are more susceptible to develop bilateral disease. The purpose of this study is to review recent literature on the topic and to highlight latest updates, regarding risk factors and possible correlations to the disease, as well as new therapeutic options.

## 1. Materials and Methods

A literature search was performed in PubMed and Google Scholar databases using the terms “bilateral herpetic keratitis”, “bilateral HSV keratitis” and “bilateral ocular herpes”. Search results were narrowed down to 86 by filtering out all articles prior to the year 2000. We also excluded all non-human studies and those written in language other than English, leaving 58 eligible. Two duplicates were also removed. After initial screening and manual evaluation of abstracts, another 7 were removed as irrelevant to the topic, describing either unilateral cases or ocular herpetic manifestations other than keratitis. The remaining 49 articles were included in the present review. Following a suggestion from our reviewers, we also included 6 additional articles to properly cite material discussed in the section regarding newer drugs in the treatment of herpetic keratitis (non-specific for bilateral cases).

## 2. Introduction

Herpetic keratitis is the result of a corneal infection with Herpes Simplex Virus (HSV) and it is recognized as a leading cause of corneal blindness worldwide [1,2]. Bilateral HSV keratitis consists of simultaneously occurring infection in both eyes.

The term “herpes” is derived from the Greek word “herpein” [pronounced: *érpin*], which means “creeping or crawling” and stands for the characteristic creeping of the eruptions caused by the virus [3].

Herpes Simplex Virus is a linear double-stranded DNA virus that is an Alpha-herpesvirinae member of the Herpesviridae family. Common pathogens in the Herpes family are Herpes Simplex Viruses 1 and 2 (HSV-1 and HSV-2), Varicella-Zoster Virus (VZV). Another common pathogen (member of Gamma herpes virus family) is Epstein–Barr Virus (EBV). All the above-mentioned viruses are neurotrophic, meaning that they have the ability to reside in a latent state within neurons of the sensory and autonomic ganglia from where they can reactivate occasionally, making the host a lifetime carrier [1].

While Herpes Simplex Virus (HSV) can infect any part of the body and elicit a variety of serious diseases in humans, it commonly infects the face, genitals and eyes with skin/face infections, typically a consequence of either oral or ocular infection [1,2]. The virus is most commonly transmitted by droplets of infected secretions, such as tears and saliva, or by direct contact of skin sores [2,4,5,6]. It was previously thought that HSV-1 had a predilection for the trigeminal ganglion and HSV-2 for the sacral ganglion [4,6]. However, ocular infection can be caused by both HSV-1 and HSV-2 [2,3]. Corneal infection is due to direct inoculation. HSV-1 may be spread to the eyes from facial sores or secretions, while HSV-2 may get transmitted either venereally, or at birth during the passage of the neonate through the vaginal tube. Conditions of crowding and poor hygiene may also facilitate transmission [5].

Primary ocular HSV infection is usually asymptomatic depending on immunological status of the host [7]. Corneal epithelial cells express specific receptors (Nectin 1, HVEM and PILR-alpha, etc.) that facilitate viral entry into the cells [5]. On contact, the virus enters the epithelial cells and starts replicating. Within hours, it enters the sensory nerve, which innervates the initial site of infection, and travels to the sensory ganglion (the trigeminal ganglion), where it may remain in a dormant state, called latency. Alternatively, it may replicate and travel back along the nerve to cause a primary infection that is clinically evident in 1% to 6% of infected patients. It can manifest as conjunctivitis, blepharitis, lid ulcers and vesicles with corneal signs seen in 33% patients. It often goes undiagnosed, especially when symptoms are mild [4,7].

Once the primary infection resolves, the virus becomes latent and stores its genome in the nucleus of a host cell, unlike retroviruses, which integrate their genome into the host’s DNA. During its latency, HSV-1 produces latency-associated transcripts (LATs) that maintain the integrity of the viral genome and reduce cellular apoptosis [8]. Later, at any point in life, certain triggers promote HSV to reactivate. The virus then utilizes the host cell’s DNA polymerase to transcribe and replicate [9], and finally it travels back down the nerve to cause a recurrent infection [4,7]. Weakening of the immune system and the presence of inflammatory mediators, such as cytokines, play an important role in the reactivation of HSV [10].

Recrudescent HSV infections are usually due to reactivation of the HSV strain acquired during primary infection. However, superinfection with a new HSV strain at the site of primary infection has also been documented [1].

## 3. Epidemiology

HSV-1 and -2 infect up to 90% of adults in the world. HSV-1 alone infects 66% of the world’s population. However, in some developing parts of the world, such as Latin America and sub-Saharan Africa, the prevalence of HSV-1 surpasses 90% [8].

Bilateral herpetic keratitis reported incidence in literature varies from 1.3% to 12% depending on the criteria used to diagnose it [7,9]. A 30-year retrospective study in the U.S. estimated the annual incidence of ocular HSV at 11.8 new cases per 100,000 of population. According to that study, 4% of all patients identified with HSV keratitis had simultaneous, bilateral involvement at initial presentation, and an additional 1% developed simultaneous bilateral involvement at the time of recurrence. Annual rates were similar between men and women. In addition, an increasing rate was recorded with aging [11]. Another large Korean study reported a 12% rate of bilateral disease among all HSV keratitis cases [12]. A study conducted in India reported a notably higher incidence of bilateral HSV keratitis, up to 25% of all herpetic keratitis cases [13]. Higher bilateral rates have also been reported in pediatric patients [14] and in patients with immunosuppression or other underlying conditions [15,16]. Table 1 summarizes the incidence rates reported in literature.

## 4. Clinical Presentation

HSV can affect all layers of the cornea. Depending on the clinical features identified on slit-lamp examination, herpetic keratitis is characterized as: Epithelial keratitis, immune or necrotizing stromal keratitis, neurotrophic keratopathy and endotheliitis, according to a recent renewal of the classification of HSK [3].

Common symptoms include redness, discharge, watery eyes, irritation, itching or foreign body sensation, and photophobia. In most patients, symptoms begin to subside after the first 2 weeks [9].

The most common subtype, epithelial keratitis, is usually due to an actively replicating virus and appears as coarse granular spots that form punctate or stellate lesions, but these quickly coalesce to form dendritic lesions. On the slit-lamp examination, epithelial keratitis presents as a dendritic lesion with terminal buds, swollen borders and intraepithelial cell infiltration. The ulcer may progressively increase in size to give a “geographical” or “amoeboid” configuration [2,5,9], especially in patients with local or systemic immunosuppression.

Stromal keratitis on physical examination appears opaque or whitened, due to stromal infiltration. Descemet membrane folds might be present as well. Corneal sensation is often reduced [13]. Similarly, the necrotizing form appears as gray-white or opaque, but there is accompanying necrosis and ulceration on slit-lamp examination. Edema and abscess may be apparent as well [2,9]. It can often result to perforation [13].

Another form of HSK, disciform lesion, has a ground-glass appearance and is disk-shaped with stromal edema (alike to different forms of endothelial keratitis) on slit-lamp examination. Neurotrophic ulcer appears with persistent central epithelial defects with a grey thickened border (Wessely immune ring) [5]. Lastly, the endothelial form appears with medium-sized keratic precipitates. Aqueous flare and cells and iritis may be visible. Stromal edema is also present [2,9,13].

Intraocular pressure could be elevated [5] when the infection causes trabecular meshwork inflammation. Bilateral herpetic keratitis presenting as peripheral ulcerative keratitis (PUK) is an extremely rare manifestation of herpetic disease. PUK can pose a diagnostic dilemma in cases with immune system disorders, such as rheumatoid arthritis. Excluding infectious agents is mandatory for appropriate treatment [16,19,20].

## 5. Diagnostic Procedures

Herpes Simplex is usually diagnosed clinically and requires no laboratory confirmation. If the diagnosis is in doubt, the following tests may assist: [5,9,21]

1. Polymerase chain reaction (PCR).
2. Scrapings of corneal or skin lesions for Giemsa stain or Tzanck smear—ELISA testing.
3. Viral culture.
4. HSV antibody titers. They rise after primary but not recurrent infection.

Immunofluorescence antibody assay (IFA) of tears.

PCR is highly sensitive, but large variation has been observed in numerous studies between the rates of HSV detection by PCR when compared to clinical diagnosis. PCR diagnostic effectiveness may be affected in patients under prophylactic anti-viral treatment with acyclovir. In addition, topical anesthetics and dyes, such as fluorescein, Bengal rose and lissamine green used for clinical diagnosis of HSV may result in decreased sensitivity of the PCR assay. The type of specimen used in detection of HSV with PCR is also an important factor. Tear specimen produces significantly less sensitive results than corneal scrapings [9]. PCR and in situ hybridization are effective and powerful techniques when other virological procedures are non-contributive, particularly in immunocompromised patients previously treated with antiviral drugs [22].

## 6. Differential Diagnosis

Misdiagnosis in clinical settings is not uncommon. Simultaneously bilateral corneal lesions resembling herpetic keratitis can be due to multiple causes such as infectious keratitis from other pathogens, post-traumatic corneal erosions, chemical injury, dystrophies, degenerations and rarely acute hydrops. Sterile corneal melt resulting from multiple eye conditions such as dry eyes, connective tissue disorder and nutritional deficiency can also result in involvement of both eyes at the same time [7,9].

Literature underscores that a significant percentage of the clinically diagnosed bilateral HSV lesions were actually caused by Varicella-Zoster Virus, adenovirus, Cytomegalovirus or Enterovirus, as confirmed by laboratory tests [9].

## 7. Risk Factors

Herpes Simplex Keratitis is, in general, a unilateral disease. Simultaneously occurring bilateral involvement is a rare phenomenon [7] and is known to develop in patients with a compromised immune system [23]. That being said, there are reports of bilateral HSV keratitis in immunocompetent patients as well [24,25]. Particularly, in another report an Acyclovir-resistant HSV variant was found to cause multiple episodes of recurrent bilateral keratitis in an immunocompetent patient [26].

In literature, bilateral Herpes Simplex Keratitis has been reported for patients with congenital immune deficiencies, atopy [27], autoimmune diseases, ocular rosacea [18], long-term immunosuppression, corticosteroid use and organ transplantation [2,23,28]. Factors such as emotional stress, fever, postoperative tear dysfunction, ultraviolet (UV-A) radiation exposure and ocular (accidental or surgical) trauma have also been related to reactivation of herpes simplex virus [13,29,30].

Below, we list a number of factors known to predispose individuals to develop bilateral herpetic keratitis. 

### 7.1. Systemic Diseases—Immunodeficiency Conditions

Rheumatoid arthritis (RA) is an autoimmune disease, characterized by an irregular immune response towards components of the connective tissue, such as collagen and elastin, also present in the structure of the cornea, and it is associated with higher incidence of bilateral HSV keratitis (40%). The characteristics of HSK in patients with RA differ from HSK in immunocompetent patients. Stromal keratitis cases were very aggressive and difficult to manage, with perforation and Gram-positive bacterial co-infection as frequently associated conditions. Prophylactic therapy at standard doses has proven unsuccessful to prevent recurrences [16]. These patients of course are usually under anti-inflammatory/immunosuppressive treatment that also favors the reactivation of HSV.

Human Immunodeficiency Virus (HIV) infection results in many cases in acquired immunodeficiency syndrome (AIDS), which is characterized by depletion of CD4+ white blood cells. A 10-year retrospective study in Tunisia reported the ocular disorders related to the disease in people living with HIV (PLWH). Most patients (86%) were on ART (Tenofovir + emtricitabine + efavirenz) combination therapy. Immunodeficiency caused by the virus increased susceptibility to opportunistic infections and neoplasms. Herpes Simplex Keratitis was found in 5% of those patients. The risk of developing ocular disease in PLWH is higher when the CD4+ count is <200 cells/μL [21].

Another category of immunocompromised patients is organ transplant recipients, as they are under long-term treatment with immunosuppressive drugs to prevent graft rejection—failure.

Cancer patients are also prone to develop bilateral herpetic keratitis. A 10-year review studied patients with malignancies that developed herpes simplex or zoster keratoconjunctivitis; 13.3% of them had bilateral disease. For the bilateral cases, the cancer diagnoses included leukemia, lymphoma/myeloma, breast and colon cancer. Only 16% of the patients were not immunocompromised or suppressed (receiving only X-ray treatment), but all others were either being actively treated for their relapsed cancer (with chemo/steroids) or immunosuppression after allogeneic stem cell transplantation (SCT) [15].

### 7.2. Age

#### 7.2.1. Childhood

There are several studies highlighting that HSV keratitis occurs more frequently as bilateral disease in the pediatric population [7,14,31,32]. The most common clinical manifestations are epithelial dendritic keratitis (38.5%) and interstitial keratitis (35.7%) [31]. Although the clinical appearance of ocular Herpes Simplex Virus (HSV) is similar in children and adults, there is evidence that stromal disease and recurrences are present in higher rates among pediatric patients [14,31,33]. Misdiagnosis of these patients is common, and therefore they are at higher risk to develop corneal scarring and opacity ultimately leading to amblyopia [14,33].

A Nigerian study in children with keratitis reported that approximately 79% of patients with HSV had combined epithelial and stromal disease. Keratitis was associated with a recent measles infection and protein calorie malnutrition. Bilateral HSV was recorded in 12.7% among herpetic cases [32].

#### 7.2.2. Senility

Increased age has been associated with reduced cell-mediated immunity, which is a crucial factor to avoid reactivation of latent viral infections, such as herpes zoster [17].

A large 30-year retrospective study in Minnesota found an age-related increased incidence in new cases of HSV keratitis, suggesting similarly the decreased immunity in elderly people as the possible cause [11].

### 7.3. Medications

Various immunosuppressive drugs have been linked to the reactivation of viral infections.

Corticosteroids are widely used as systemic treatment in a plethora of indications, including autoimmune diseases and atopy, to mitigate the immune system’s response and reduce inflammation. A latent HSV infection could be reactivated by rapid tapering of systemic corticosteroids, as both steroid use and rapid tapering may act as a triggering factor for viral infection or reactivation of herpes [23].

Rituximab is a B cell depleting anti-CD20 monoclonal antibody that is increasingly used to treat autoimmune disorders and B cell non-Hodgkins lymphoma. As with other immunosuppressive agents, there is the risk of opportunistic infections or reactivations, including Hepatitis-B virus and Herpes viruses (HSV). There is a rare case report of a patient suffering from severe mucous membrane pemphigoid treated with rituximab who developed herpetic keratitis. A 71-year-old patient suffered from severe mucous membrane pemphigoid (an autoimmune blistering disease affecting predominantly the mucosae) with ocular, oral pharyngeal and laryngeal involvement. To control the disease, the patient was given rituximab therapy in combination with oral corticosteroids. He subsequently experienced an epithelial herpes simplex virus keratitis in one eye and 3 months later in his other eye [34].

Ocular anti-hypertension drugs and prostaglandin analogs. There is a strong debate in literature about the role of anti-glaucoma drugs and prostaglandin analogs, especially in triggering recurrent herpetic keratitis.

Several case reports suggested a causal relationship between the use of topical prostaglandin eye drops and episodes of bilateral HSV keratitis. Both latanoprost [35], travoprost [2] and bimatoprost [36,37] were thought accountable. Authors support their hypothesis with the observation that all incidents happened during the use of anti-glaucoma drops and that the patients had no other predisposing factors to explain recurrence of keratitis [1]. Moreover, by administrating anti-viral agents patients recovered within days [36] and by discontinuing the suspected drug there were no recurrences of keratitis [35]. Additionally, in some cases, when the patient resumed treatment with the same drug, bilateral keratitis re-occurred [2,36] with increased severity [37]. Antiglaucoma prostaglandin analogues induce the release of endogenous prostaglandins in the iris and the ciliary muscle, acting as inflammation mediators. This mechanism probably explains the reactivation of HSV keratitis [36]. All these reports share a common weakness: Diagnosis of HSV relied on clinical findings only, without laboratory confirmation. 

Some authors, however, acknowledge that corneal toxicity from the preservative contained in eye drops may have contributed to herpes’ reactivation [2]. In addition, a recent case series study emphasizes that corneal toxicity is commonly misdiagnosed by physicians as herpetic keratitis, due to the similar appearance of corneal lesions. This study included patients under treatment with all categories of anti-glaucoma drops, initially referred with presumed diagnosis of HSK. Half of them had bilateral corneal lesions. Most drops in the study also contained benzalkonium (BAK) as a preservative. Daily and repetitive exposure of ocular surface to the active compounds and the preservatives in the topical anti-glaucoma medications is often reported to cause toxic effects to the ocular surface. Furthermore, both HSK and chronic use of topical medications containing BAK can cause impaired corneal sensation [38].

Corneal toxicity usually appears as punctate epithelial defects, which over time collide to form linear or branch-like lesions resembling the characteristic herpetic dendritic lesions. The main distinctive details are that the pseudodendritic lesions have rather heaped-up edges, they lack terminal buds and they are usually surrounded by diffuse punctate defects. The authors using specific clinical criteria determined that their patients’ lesions were not actually related to HSV and confirmed their findings with negative viral cultures. All patients recovered after removal of the presumed triggering medication or by applying ocular surface protecting methods, including topical lubricants or therapeutic soft contact lens, and without the need of anti-viral treatment [38].

## 8. Rare Cases

Recent literature reports describe rare or unique cases of certain conditions or surgical procedures that were observed along with incidents of bilateral herpetic keratitis, indicating a possible causal relationship.

### 8.1. Cases Reports Involving Autoimmune Diseases

Rare case of pemphigus foliaceus complicated by Kaposi’s varicelliform eruption and bilateral HSV keratitis: Pemphigus foliaceus (PF) is a chronic autoimmune blistering dermatosis (ABD) that often involves the face, scalp and trunk. Kaposi’s varicelliform eruption (KVE) is an uncommon widespread skin viral infection over some pre-existing skin disease with variable severity that rarely complicates ABD, and it is most predominantly caused by herpes simplex virus (HSV) infection. In addition, KVE infrequently causes ocular involvement including Herpes Simplex Keratitis (HSK) presented with large geographic corneal lesions. Timely diagnosis of KVE may be difficult in patients with PF due to their overlapping clinical features. PF complicated with KVE and HSK is extremely rare, and there is only one such case reported up to date. Therefore, it is important to raise high suspicion of HSV infection in patients with underlying ABD if recurrence or resistance to treatment occurs, especially when combined with ocular symptoms [2].

Another similar case report described a patient with PF treated with azathioprine and high-dose corticosteroids developing bilateral HSV keratitis following rapid tapering of steroids, which is known to trigger Herpes reactivation [23].

In a third case, a young man with chronic atopic dermatitis developed Kaposi’s varicelliform eruption and simultaneous onset, bilateral HSV-1 (confirmed by direct immunofluorescence) dendritic epithelial keratitis with corneal epithelial edema during a generalized dermatitis incident [27].

### 8.2. Pregnancy

A young woman developed bilateral HSK during pregnancy. Her ocular history included use of contact lenses. Pregnancy can resemble a modified state of immunosuppression resulting in an increased risk for HSV keratitis. This combined with contact lens wear may have an additive effect and increase the possibility of HSV recurrence [39].

### 8.3. Lactation

A young lactating woman with a clear medical history presented simultaneous bilateral keratitis that was confirmed to be herpetic with PCR. The authors suggest that lactation in this case may have aggravated malnutrition and anemia leading to a certain immune susceptibility, allowing a latent herpetic infection to reactivate. Even though the virus can spread from mother to child through direct contact with infected secretions (i.e., saliva or tears) or skin and mucosal lesions, breast feeding is not contraindicated in any active herpetic infection unless lesions are on the breast [7].

### 8.4. Neonates

A premature baby delivered by cesarean section at 26 weeks of gestation. Mother had no history of HSV or signs of active disease at the time of birth. Nine days later the newborn child developed facial vesicle and bilateral eyelid edema. Examination revealed corneal epithelial lesions also. There was also central nervous system (CNS) involvement. Laboratory workup showed HSV-1 infection. At the time of diagnosis, maternal serology was also negative, indicating first-episode primary infection. It is important to highlight that half of the infants with HSV infection are born prematurely, usually between 30 and 37 weeks of gestation [40].

In another case, a newborn delivered vaginally developed herpetic vesicular rash and bilateral corneal ulcers. In this instance, HSV-2 was identified by direct immunofluorescent staining of vesicle fluid. Many neonatal infections occur because of asymptomatic cervical shedding of virus, usually after a primary episode of HSV infection. The infant is protected for the first few months of life by circulating maternal antibodies, but this might not be the case always [41].

### 8.5. Graft-Versus-Host Disease (GVHD)

Although rare, bilateral herpetic keratitis has been reported in patients with GVHD. In a case report an 11-year-old boy underwent allogeneic bone marrow transplantation as treatment for myelocytic leukemia. Three months post-transplantation he developed symptoms of the skin, eyes and mouth, and a lip biopsy indicated chronic graft-versus-host disease (GVHD). GVHD is a major complication of allogeneic bone marrow transplantation that occurs due to the reactivity of transplanted immunocompetent cells against host cells. The child had a persistent keratitis with corneal filaments and neovascularization in both eyes and received long-term treatment with fluorometholone eyedrops among other medication. He later developed bilateral Herpes Simplex Keratitis with geographic ulcers, but responded well to acyclovir ointment [42].

### 8.6. Viral Infections

Viral conjunctivitis: Bilateral disciform keratitis is a late complication of viral conjunctivitis. A young man in the course of viral conjunctivitis developed bilateral disciform keratitis in the span of 3 weeks with widespread sub-epithelial corneal infiltrates in addition to a central corneal edema with a white distinct border resembling Wessely ring, as well as Descemet’s folds and keratic precipitates in the central area. The patient was treated with both oral and topical antiviral, and showed significant improvement. Herpetic disciform keratitis is a primary endotheliitis resulting in both stromal and epithelial edema in a round (disciform) distribution with keratic precipitates underlying the area of edema [4].

COVID-19: Continuing research on the novel SARS-CoV2 virus brings to light more and more of the virus’ wide spectrum of clinical features and correlations. 

A recent Slovakian study noted that during the first pandemic wave, a 2.5- and 2-fold higher incidence of herpetic keratitis was recorded in the area of the study in comparison to the same period in 2019 and 2018, respectively. It is known that viral reactivation may occur in critically ill patients or due to a weakened immune system, fever or hormonal changes. COVID-19 patients undergo immunosuppression and cytokine storm syndrome. These characteristics are mostly found among critically ill patients. T lymphocytes and natural killer cells decrease in number and become functionally exhausted, particularly CD8+ T cells. These cells are responsible for the control of viral infections. HSV-1-specific CD8+ T cells keep herpes in a latent state. The exhaustion of these cells leads to impaired effector function and may allow the reactivation of HSV-1 that resides in the trigeminal ganglion. According to these mechanisms, COVID-19 may be a potential activator of HSV-1 infection [10].

### 8.7. Ocular Surgery

Refractive laser surgery: Only a few human cases of Herpes Simplex Keratitis associated with Laser in Situ Keratosmileusis (LASIK) and Photo-Refractive Keratectomy (PRK) have been reported. Chao-Kung Lu et al. described a case of simultaneous bilateral HSK one month after uneventful LASIK surgery for correction of myopia in an otherwise healthy woman without prior history of herpes. The dendritic lesions appeared on the corneal flap in both eyes. Laser irradiation may have triggered reactivation of a latent previously asymptomatic HSV infection [29].

A similar effect can result from excimer laser photokeratectomy (PTK). A PCR assay detected viral DNA in tears’ specimen postoperatively. It is suggested that the procedure may stimulate viral reactivation and consequent shedding in the tear film [43].

Corneal collagen crosslinking (CXL) utilizes ultraviolet radiation to enhance corneal collagen bonding in order to stabilize progression of keratoconus. Exposure to ultraviolet A (UVA) light induces the secretion of interleukine IL-10, which suppresses the immunological response against infectious agents. Therefore, CXL treatment could be a stimulus to trigger reactivation of latent HSV infections even in patients with no history of clinically evident herpes virus ocular infections. Among the various complications of this procedure, recrudescence or a first episode of viral keratitis has been reported in literature. An interesting case report presented a young immunocompetent patient treated with CXL in both eyes at the same setting (due to social reasons—the patient lived in a distant location) for bilateral progressive keratoconus. Shortly after surgery he developed clinical signs of bilateral HSV keratitis. The authors acknowledged that the decision to treat both eyes together resulted in this rare manifestation. Therefore, they suggested that the optimal choice is to avoid simultaneous application of CXL and that prophylactic administration of anti-virals may be beneficial [30].

Corneal transplantation: A case series study included 30 patients that suffered from recrudescent herpetic keratitis, with 1 of them having bilateral disease. Researchers separated these patients into two groups, depending on whether the patients underwent penetrating keratoplasty (PKP) or not, and they compared the HSV genotype identified with a PCR assay from specimens obtained during the follow-up period, between sequential episodes of keratitis, both prior and after transplantation. Analysis of the collected data showed that many of the patients in the transplantation group had been superinfected with a different HSV strain, while in the other group, all HSK recurrences were due to the original HSV strain. Their findings suggested that PKP may be a risk factor for transmission of HSV-1 with subsequent reactivation of the donor-derived HSV-1 strain in the corneal allograft colonized the recipient. The DNA sequences were identical in both strains isolated from the transplant and the recipient, providing conclusive evidence for graft-to-host transmission of HSV-1 through corneal allograft [1].

Botulinum toxin injection: A 59-year-old immunocompetent woman with functional epiphora was transconjuctivally injected with Botulinum toxin-A into each lacrimal gland. Her medical history included HSV keratitis quiescent for the previous 2 years. Three weeks after the injection she developed bilateral stromal keratitis, suggesting that the procedure may have triggered reactivation. The authors highlighted the need for oral antiviral prophylaxis before BTA injections [44].

Strabismus surgery: Another case report study described a 3-year-old child who underwent bilateral lateral rectus recessions to correct strabismus. She was prescribed with standard post-operative dexamethasone and antibiotic eye drops. One week later the child presented fever along with initially unilateral eyelid swelling and ptosis, which within days progressed in the other eye as well. Clinical examination showed bilateral corneal erosions confirmed to be herpetic by PCR. It is apparent that both the surgery and the use of steroids contributed in this case [45].

Intravitreal anti-VEGF injection: A man suffering from exudative Age-related Macular Degeneration (wet –AMD) had been uneventfully under treatment for approximately 4 consecutive years with multiple intravitreal injections of triamcinolone and antivascular endothelial growth factor agents, as well as Photo Dynamic Therapy (PDT), in both eyes. Then, just days after receiving treatment with bevacizumab injection in one eye and a triple combination of intravitreal bevacizumab, triamcinolone, plus full-fluence PDT in the other eye (both in the same session), the patient developed bilateral dendritic keratitis. He then reported a history of mouth sores arising periodically, with the most recent episode at the time of the injections [46]. 

The authors mentioned that the patient continued treatment with anti-VEGF injections bilaterally with no recurrence of HSV keratitis or other complications up to publication date. They also suggested that his history of long-standing diabetes mellitus, if poorly regulated, may have posed a vulnerability contributing to the reactivation of herpes. Of course, the presence of active mouth sores during the time of the ocular injections implies that a generalized herpetic eruption was emerging and along with the ocular manipulations they possibly triggered reactivation of keratitis as well [46].

Trabeculectomy with Mitomycin-C (MMC): Finally, a short case series describes 3 patients who presented clinically diagnosed herpetic keratitis after trabeculectomy with use of the antimetabolite MMC. One of them developed bilateral keratitis. According to the authors, the time interval between the glaucoma surgery and the occurrence of HSV keratitis ranged from 15 days up to 2 years post-operation. They suggested that mitomycin may facilitate development of herpetic keratitis/keratouveitis up to 2 years after glaucoma surgery [47].

## 9. Treatment

### 9.1. Medical Treatment

Most infections of herpetic keratitis are self-limiting, even without treatment. However, it is essential to treat the infection at the earliest onset to reduce viral replication, shorten disease course and maintain latency in order to prevent further complications. That being said, existing treatment reduces the severity of lesions and controls further viral spread but does not provide a permanent cure. Recurrences can still happen, despite treatment with antiviral drugs [9].

Treatment depends on the type of corneal lesions. Epithelial keratitis can be treated with topical anti-virals only. Corneal debridement under local anesthesia is also beneficial. In immunocompromised patients, patients poorly responding to topical therapy and wherever we need to avoid corneal toxicity associated with certain topical agents such as triflurothymidine drops, the addition of oral anti-virals are suggested. If high intraocular pressure occurs, anti-glaucoma drops may be administered, except for prostaglandin analogues, as they promote viral activity and inflammation in general [5].

Whenever there is stromal involvement, as in cases of disciform, neurotrophic and necrotizing keratitis, topical steroids should be administered along with anti-virals. Gradual tapering of both medications is recommended upon improvement [5]. 

Current treatment for HSK includes acyclovir, ganciclovir, triflurothymidine, penciclovir and valacyclovir. Acyclovir and its derivatives are nucleoside analogs [9].

Acyclovir has a few downsides as treatment of ocular HSV-1 infections. The first is that it affects only newly synthesized viral DNA, and therefore it does not cure infected cells of the virus, but it does prevent new viruses from being produced [9]. Secondly, acyclovir is susceptible to drug resistance. Many cases of drug resistance have been reported, and immunocompromised patients appear especially vulnerable to developing resistant HSV-1 infections [8,9,26]. In addition, acyclovir has poor bioavailability, so that high doses and increased frequency of administration are required [9]. 

On the other hand, valacyclovir, a prodrug of acyclovir, has better bioavailability than oral acyclovir and produces higher acyclovir tissue and serum concentrations. Topical ganciclovir has been shown to be as safe and effective as acyclovir in the treatment of herpetic epithelial keratitis [48]. Furthermore, topical ganciclovir can reach therapeutic levels in the cornea and aqueous humor following topical application. Despite that, prolonged use of thymidine analogs may lead to toxicity of the ocular surface, including epithelial keratitis, corneal ulcers, follicular conjunctivitis, and punctal occlusions [48].

Second-line treatment includes foscarnet and cidofovir, but they have less specificity for viral DNA and are more likely to cause significant toxicity in patients [9,26]. Cidofovir has a different mechanism of action than acyclovir and it has been used to cure immunocompromised patients with acyclovir-resistant HSV, allowing them to be treated again with acyclovir when their original acyclovir-susceptible HSV strain reactivated again.

### 9.2. Surgical Treatment

Penetrating Keratoplasty (PKP) is the last option when the disease causes irreversible corneal damage, scarring or melting. However, recurrence of the disease or rejection may threaten the survival of grafts [1,5,19].

### 9.3. Prophylactic Therapy

Long-term administration of low-dose oral antivirals as prophylaxis has demonstrated a significant decrease in recurrence of all forms of herpetic eye disease [11,23]. The HEDS study and other recent studies have shown that 400 mg of oral acyclovir twice daily reduced the 1-year ocular HSV recurrence rate by approximately 45% [12]. Additionally, patients treated with oral antivirals for only 12 months showed higher rates of recurrence and shorter disease-free intervals as opposed to those treated longer than 12 months, suggesting a benefit of treatment with oral acyclovir beyond 1 year [11].

Moreover, the addition of oral ascorbic acid in prophylactic treatment was associated with further reduced risk of HSK recurrence [12].

Currently approved drugs that are active against most ACV resistant mutants have major limitations in terms of toxicities and pharmacokinetic liabilities that prevent their prophylactic use [26].

Another study, though, regarding patients with rheumatoid arthritis and bilateral herpetic keratitis showed that prophylactic therapy at standard doses did not offer any benefit in avoiding recurrences, highlighting, however, the need for intense monitoring [16].

### 9.4. Development of New Anti-Viral Drugs

Newer drugs are being tested as alternatives for the treatment of HSV. These molecules target at different phases of HSV-1 infection: Aptamers, retrocyclin 2 and antibodies act against viral attachment [49,50]; G1, G2 and other cationic peptides obstruct entry into the host cells [51]; nucleoside analogs and CRISPR/Cas9 inhibit DNA replication [52]; BX795 block protein synthesis [53]; and OGT 2115 restrains egress [54]. These therapies demonstrated significant antiviral effects during their use in laboratory in vitro, in vivo, and ex vivo experiments. BX795 and OGT 2115 look quite promising against resistant to acyclovir HSV variants [53,54]. The HSV-1 strain would have to acquire resistance to three different mechanisms of treatment in order to replicate and infect other cells successfully [8].

## 10. Discussion

Simultaneously occurring bilateral herpetic keratitis has a high proportion of subsequent complications, either because of greater virulence of the virus or because of altered host susceptibility [7]. Therefore, prompt administration of medication upon diagnosis is essential to reduce the severity and the frequency of viral attacks and delay progression of corneal damage. This is highly important in children, because bilateral scarring and opacification may result in permanent amblyopia, with devastating outcomes in functional vision [14].

Patients at greater risk for recurrences should be treated long-term with prophylactic doses of anti-virals. In addition, as mentioned above, there are numerous ocular surgical procedures that may trigger herpetic reactivation. Consequently, it may be beneficial to administrate prophylactic treatment to these patients prior to operations.

The role of prophylaxis should be reconsidered for another reason as well. As studies have shown, herpetic infection promotes serious structural changes and immunological response in the cornea. Confocal microscopy revealed that these effects are in fact bilateral, even in apparently unilateral cases [3,55].

Acyclovir resistance is a growing concern, especially in patients with an affected immune system. Hopefully, the development of new anti-viral agents will address this issue. Molecules on trial, used in combination, have shown effectiveness against resistive HSV variants by targeting different stages in the viral life cycle [8].

Studies from developing countries tend to report a higher incidence of bilateral herpetic keratitis [13,32]. Another study noted that even in a wealthy country, people living under poverty show nearly a 2-fold rate of HSV infections compared to higher-income individuals [8]. The fact that overcrowding conditions and poor hygiene facilitate the spread of HSV may explain the difference. Malnutrition may contribute as well [5,32]. Another possible reason may be a limited or delayed access to health services.

## Figures and Tables

**Table 1:** Incidence of bilateral HSV keratitis in previous studies.

Study	Setting	Incidence of Bilateral HSV Eye Cases	Description of Bilateral HSV Keratitis Cases
Ref. [12] Clinical Features of Herpes Simplex keratitis in a Korean Tertiary Referral center. Efficacy of Oral Antiviral and Ascorbic Acid on Recurrence	Retrospective study from January 2010 to January 2015 at Gyeongsang National University Hospital in Jinju, South Korea	16/133 patients 12.0%	The patients were followed for 24.1 ± 13.2 months on average The mean age of onset was 61.1 ± 15.6 years The mean BCVA at onset was 0.65 ± 0.63
Ref. [17] Ocular involvement and visual outcome of Herpes Zoster ofthalmicus: review of 45 patients from Tunisia, North Africa	Retrospective study from January 2000 to January 2012 Department of Ofthalmology at Fattouma Bourguiba Hospital of Monastir, Tunisia	6 of 45 patients 13.3%	2 of them had diabetes 2 were under immunosuppressive therapy 1 had HIV infection and the past medical history was remarkable in the remaining 1 patient
Ref. [11] The Incidence, Recurrence and Outcomes of Herpes Simplex Virus Eyes Disease in Olmsted County, Minnesota, 1976 through 2007: the impact of Oral Antiviral Prophylaxis	Retrospective study from 1976 to 2007 at Olmsted County, Minnesota	20 of 394 patients 5%	16 of them had bilateral involvement at initial presentation 4 additional patients had bilateral involvement at the time of recurrence
Ref. [1] Corneal Herpes Simplex Virus Type I Superinfection in patients with Recrudescent Herpetic Keratitis	Rotterdam Eye Hospital, Rotterdam The Netherlands	1 of 30 patients 3.33%	In this patient the bilateral herpetic keratitis was due to infections with different HSV-1 strains in either cornea
Ref. [13] Clinical profile of Herpes Simplex Keratitis cases attending eye Opd in tertiary hospital Chhattisgarh state	Prospective study from March 2016 to February 2017 at the Department of ophthalmology, Government Medical College, Rajnandgaon, India	20 of 80 patients 25%	In a series of 356 patients over 30 years in Japan bilateral keratitis was found in 9.4%. This may be due to overall increased incidence of the disease. Bilateral cases are mainly epithelial keratitis which complies with other study.
Ref. [15] Bilateral Herpetic Keratocunjunctivitis in cancer patients	Retrospective study from June 2001 to August 2011 at MD Anderson Cancer Center	12 of 90 patients 13.3%	5 of them were in remission from their cancer and the other 7 were in active cancer treatment Only 2 patients were not immunocompromised or suppressed 7 of the patients were on systemic steroids and the other 5 were on prophylactic anti-viral medication at the time of presentation. 1 of them had disseminated herpetic disease and was on IV antiviral therapy
Ref. [16] Herpes Simplex Keratitis in Rheumatoid Arthritis Patients	Retrospective study	2 of 5 patients 40%	
Ref. [14] Herpes simplex virus keratitis in children	Retrospective cohort study	6 of 23 patients 26%	
Ref. [18] Bilateral herpetic keratoconjunctivitis	Retrospective study from January 1996 to September 2001 at the Department of Opthalmology, University of Minnesota	7 of 544 patients 1.3%	5 of these patients had systemic atopy and the other 2 ocular rosacea Systemic immune disorders were noted in two patients. Recurrent blepharoconjunctivitis was noted in 8 eyes (57%), epithelial keratitis in 12 eyes (85.7%) stromal keratitis in 9 eyes (64.3%) necrotizing stromal keratitis in 5 eyes (35.7%) progressive endotheliitis in 2 eyes (14.2%). Penetrating keratoplasty was performed in 1 eye, in which endophthalmitis subsequently developed and which required enucleation.
